# Investigation of the role of the miR17-92 cluster in BMP9-induced osteoblast lineage commitment

**DOI:** 10.1186/s13018-021-02804-9

**Published:** 2021-10-30

**Authors:** Yunyuan Zhang, Xuran Jing, Zhongzhu Li, Qingwu Tian, Qing Wang, Xian Chen

**Affiliations:** 1 Department of Clinical Laboratory, The Affiliated Hospital of Qingdao University, Qingdao, Shandong China; 2 Department of Molecular Laboratory, Qingdao, Endocrine and Diabetes Hospital, Qingdao, Shandong China; 3 Department of Clinical Laboratory, Pingyi Hospital of Traditional Chinese Medicine, Linyi, 273300 Shandong China

**Keywords:** miR 17-92, BMP9, Osteogenesis, *Rb*

## Abstract

**Background:**

Bone morphogenetic protein 9 (BMP9) has been identified as a crucial inducer of osteoblastic differentiation in mesenchymal stem cells (MSCs). Although microRNAs (miRNAs) are known to play a role in MSC osteogenesis, the mechanisms of action of miRNAs in BMP9-induced osteoblastic differentiation remain poorly understood.

**Methods:**

In this study, we investigate the possible role of the miR17-92 cluster in the BMP9-induced osteogenic differentiation of MSCs by using both in vitro and in vivo bone formation assays.

**Results:**

The results show that miR-17, a member of the miR17-92 cluster, significantly impairs BMP9-induced osteogenic differentiation. This impairment is effectively rescued by a miR-17 sponge, an antagomiR sequence against miR-17. Using TargetScan and the 3′-untranslated region luciferase reporter assays, we show that the direct target of miR-17 is the retinoblastoma gene (RB1), a gene that is pivotal to osteoblastic differentiation. We also confirm that RB1 is essential for the miR-17 effects on osteogenesis.

**Conclusion:**

Our results indicate that miR-17 expression impairs normal osteogenesis by downregulating RB1 expression and significantly inhibiting the function of BMP9.

**Supplementary Information:**

The online version contains supplementary material available at 10.1186/s13018-021-02804-9.

## Introduction

Mesenchymal stem cells (MSCs) are multipotent progenitor cells with the capacity to self-renew and the ability to differentiate into osteoblastic, adipogenic, myogenic, and chondrogenic lineages [1–5]. Because of their ease of isolation and distinctive characteristics, MSCs have been widely regarded as potential candidates for tissue engineering and regenerative medicine [6, 7]. Osteogenic differentiation of MSCs is a complex process that is tightly regulated by key molecules and several signaling pathways [8, 9]. One such key regulator is a group of growth factors known as bone morphogenetic proteins (BMPs). BMPs, members of the TGF-β superfamily, are critical regulators of skeletal development and MSC differentiation [10–13]. Specifically, BMP9 has been identified as a crucial inducer of osteoblastic differentiation in MSCs [12, 14]. However, the specific pathways involved in the BMP9-induced osteoblastic differentiation remain poorly understood.

Recent research has focused on the role of microRNAs (miRNAs) in osteogenesis [15–17]. miRNAs are a group of evolutionarily conserved, endogenous, small (~ 17–24 nucleotide) non-coding RNAs (ncRNAs) [18]. Over the last decade, there have been significant insights into the molecular biology of miRNAs and their function in RNA silencing[19–21]. By inducing targeted mRNA degradation and sequestration, miRNAs inhibit protein translation and thereby regulate gene expression [22].

In this study, we investigate the possible role of the miR17-92 cluster in the BMP9-induced osteogenic differentiation of MSCs. The miR17-92 cluster is located within the third intron of the open reading frame 25 (*C13orf25*) and encompasses six miRNAs (miR-17, miR-18a, miR-19a, miR-20a, miR-19b-1, and miR-92a-1) [23]. Dysregulation of the miR17-92 cluster has been linked to signaling pathways such as Wnt/β-catenin, NF-κB, and TGF-β that are involved in a number of developmental diseases [24]. However, the role of the miR17-92 cluster in osteogenesis is unclear.

Here, using both in vivo and in vitro studies, we demonstrate that miR-17, a member of the miR17-92 cluster, significantly impairs BMP9-induced osteogenic differentiation. This inhibition is effectively rescued by a miR-17 sponge, an antagomiR sequence against miR-17 that silences the endogenous miRNA. Using TargetScan and 3′-untranslated region (UTR) luciferase reporter assays, we determine that the direct target of miR-17 is the retinoblastoma gene (*RB1*), a gene that is pivotal to osteoblastic differentiation [25].

Our results demonstrate that miR-17 expression impairs normal osteogenesis by downregulating *RB1* expression and suppressing the function of BMP9. These findings could lead to an innovative approach to bone regeneration where anti-miRNA therapies could be devised to inhibit miR-17 and increase the osteogenic differentiation of MSCs for bone repair.

## Materials and methods

### Cell culture and adenoviral infection

Immortalized mouse embryonic fibroblasts (iMEFs), well-characterized as MSC lines, were isolated from CD1 mice on days 12.5–13.5 post-coitus and characterized as previously described [24]. HEK293 cells (purchased from ATCC, Manassas, VA) were cultured in complete Dulbecco's Modified Eagle’s Medium (DMEM, HyClone, Logan, UT) supplemented with 10% fetal bovine serum (FBS, Gibco, Grand Island, NY), 100 U/ml of penicillin, and 100 μg/ml of streptomycin (Solarbio, China). The cells were incubated at 37℃ in 5% CO_2_ and stimulated with BMP9 adenovirus (AdBMP9) to induce osteoblast differentiation. Stable iMEF lines expressing each member of the miR17-92 cluster were established using 4 μg/ml blasticidin selection over a period of 7 days.

For the osteogenic differentiation experiments, cells were induced in osteogenic media containing BMP9 supplemented with 100 nM of dexamethasone (Solarbio), 0.2 mM of ascorbic acid (Solarbio), and 10 mM of β-glycerophosphate (Sigma-Aldrich, USA).

### DNA constructs

All the PiggyBac vector constructs used in this study were based on the pMPB-omiR vector (http://www.boneandcancer.org/ucmolab.html). miR-17 (miRBase accession number: MI0000687), miR-18a (miRBase accession number: MI0000567), miR-20a (miRBase accession number: MI0000568), and miR-92a-1 (miRBase accession number: MI0000719) overexpression vectors were constructed using miRNA-5p fragments. Annealed miR-17 sponge fragments were constructed into pMPB-omiR, hereinafter referred to as Sponge-miR-17.

The potential target genes of miR-17 were predicted using miRWalk 2.0. Segments of the 3′UTRs of *RB1* and *BMPR2* mRNA were PCR-amplified from mouse genomic DNA. The resulting DNA was then subcloned into the *Sac*I and *Hind*III sites of the pMIR firefly luciferase reporter vector (Promega). All the plasmids were sequenced to ensure authenticity.

### RNA isolation and touchdown quantitative real-time PCR (TqPCR)

Total RNA was isolated using TRIzol Reagent (Invitrogen, Carlsbad, CA) and purified with a DNA-free RNA kit (Zymo Research Corporation, Irving, CA) according to the manufacturer’s instructions. The RNA was then reverse transcribed using hexamers and M-MuLV Reverse Transcriptase (New England Biolabs, Ipswich, MA). The resulting complementary DNA products were diluted 10- to 100-fold and used as PCR templates. Quantitative PCR analysis was carried out using our optimized TqPCR protocol. Briefly, SYBR Green qPCR (Bimake, Houston, TX) reactions were set up according to the manufacturer’s instructions. The cycling program was modified by incorporating 4 cycles of touchdown steps prior to the regular cycling program described in the manufacturer’s instructions. To determine the expression levels of miR-17, TaqMan MicroRNA assays directed to mmu-miR-17 were performed using the manufacturer’s protocol. U6 and GAPDH were used as internal references, and all sample values were normalized to U6 or GAPDH expression by using the 2^−ΔΔCt^ method.

### Dual luciferase assays

The iMEF cells were seeded in 24-well cell culture plates 24 h before transfection and then co-transfected with 50 ng of luciferase vector containing either the 3′UTR of *RB1*, the 3′UTRs of *BMPR2*, *SMAD7*, miR-17, or their respective controls. Forty-eight hours post-transfection, equal numbers of cells were lysed, and luminescence was measured according to the manufacturer’s instructions. *Renilla* was used as a control.

### Alkaline phosphatase (ALP) assays

After BMP9-stimulation for 3 to 7 days, alkaline phosphatase (ALP) staining was performed on the iMEFs, as previously described [26]. Briefly, the cells were washed 3 times with PBS and fixed with 0.05% glutaraldehyde at room temperature for 10 min. The cells were then stained with a mixture of 0.1 mg/ml of naphthol AS-MX phosphate and 0.6 mg/ml of Fast Blue BB salt. After 20 min, the mixture was removed and replaced with PBS. Histochemical staining was recorded using bright field microscopy.

### Alizarin red S staining and quantification

The iMEFs were stimulated for 14 days with BMP9 and detected as previously described [27]. The cultured cells were then fixed with 1% glutaraldehyde and then stained with 2% Alizarin red S (Sigma-Aldrich) for 20 min at room temperature. The cells were monitored under a microscope every 2–5 min during the staining process. The staining of calcium mineral deposits was then recorded using bright field microscopy.

### Xenograft mouse model

All animal procedures were approved by the Animal Care Committee of the Affiliated Hospital of Qingdao University. iMEFs were cultured with BMP9 for 24 h before the in vivo study. Six-week-old NOD/SCID mice (obtained from Weitong Lihua Experimental Animal Technology Co., Ltd, Beijing, China) were randomly divided into 5 groups. General anesthesia was administered via intramuscular injection of pentobarbital sodium (0.1 ml/100 g) for all surgical procedures. For a single transplant complex, 5 × 10^5^ cells were washed with PBS and subcutaneously implanted into the back of the mice. After 4 weeks, the mice were sacrificed by cervical dislocation under general anesthesia. The implants were then harvested, fixed with 1% formaldehyde, and decalcified for 48 h. For histological analyses, the implants were embedded in paraffin, sectioned and stained with H&E and Masson’s trichrome stain (Solaibao Biotechnology Co., Ltd, Beijing, China) according to the manufacturer’s instructions.

### Statistical analysis

All data were presented as the mean ± standard deviation from at least three independent experiments, and differences between groups were analyzed using a two-tailed unpaired Student’s t-test. A *p* value of < 0.05 was considered statistically significant.

## Results

### MiR-17 is significantly downregulated during BMP9-induced osteogenic differentiation

**Fig. 1:**
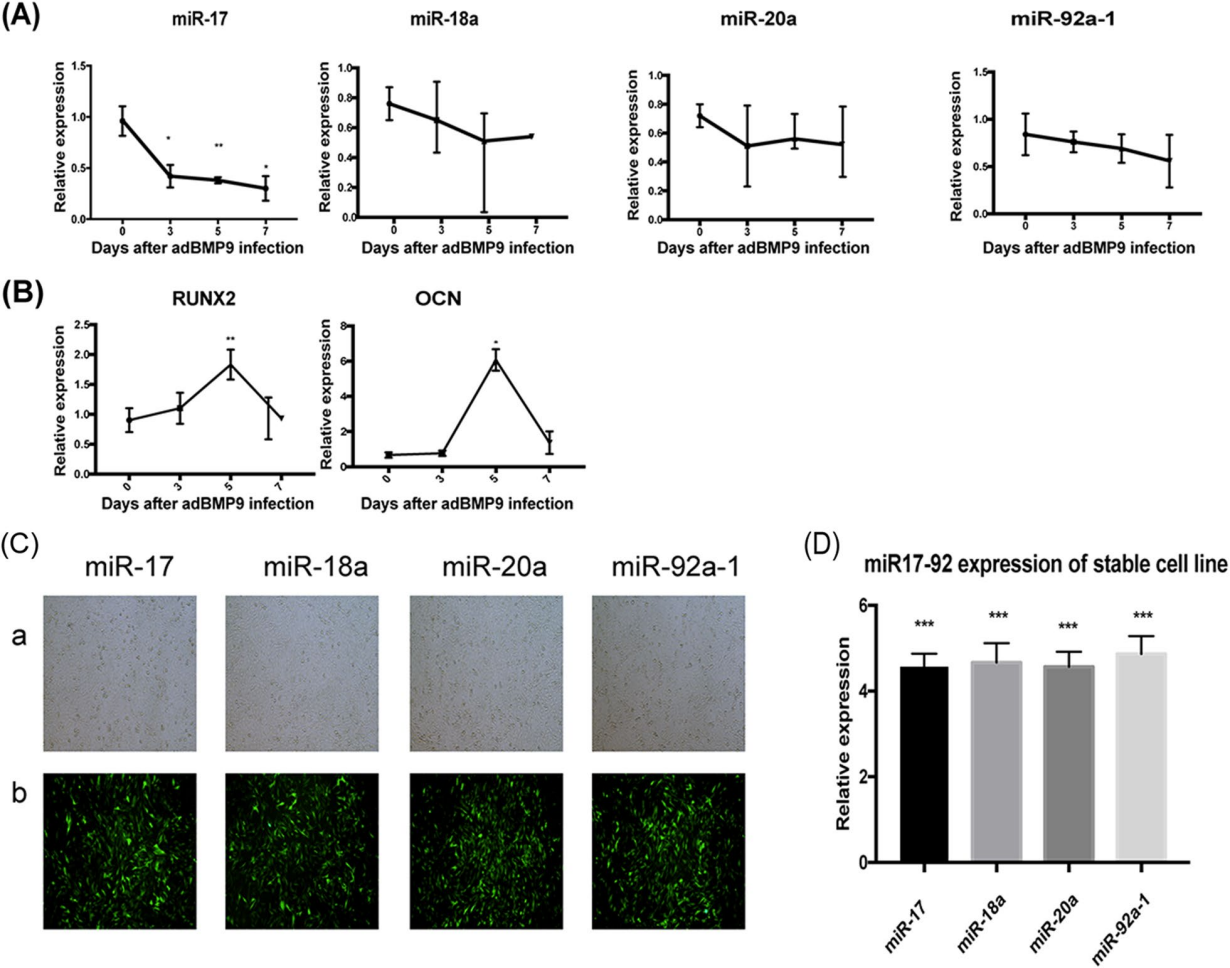
Expression levels of individual member of miR-17-92 and osteogenesis markers in BMP9-induced MSCs. **A** The expression levels of mouse miR-17, miR-18a, miR-20a and miR-92a-1 in BMP9-induced iMEFs at the indicated time points. **B** The expression levels of osteogenesis markers *OPN* and *OCN* at the indicated time points. Samples were normalized with the reference gene *gapdh*. Each assay condition was done in triplicate. **p < 0.01, *p < 0.05. *Runx2*, runt-related transcription factor 2; *OCN*, osteocalcin. **C** Stable cell lines expressed individual member of miR-17-92 cluster. (a) Blank fields of stable cell lines. (b) Fluorescence fields of stable cell lines. **D** qPCR detected the expression of individual member of miR-17-92 cluster. All samples were normalized with the reference gene *gapdh*. Each assay was done in triplicate. *p < 0.05, **p < 0.01

To determine the role of the miR-17-92 cluster in the osteogenic differentiation of MSCs, we used TqPCR to analyze the expression of miR-17-92 cluster members in iMEFs (our MSC lines). Our results showed that miR-17 expression dramatically decreased from day 3 to day 7 in BMP9-induced iMEFs compared to the miR control group (hereinafter referred to as miR Control). In contrast, there was no significant decrease in the expression of either miR-18a, miR-20a, or miR-92a-1 (Fig. 1A). These results suggest that miR-17 plays an important role in BMP9-induced osteogenic differentiation.

We then assessed whether there was a correlation between the expression of miR-17 and the expression of osteogenic regulators and osteogenic markers. Runt-related transcription factor 2 (Runx2), an early differentiation factor expressed in precursor osteoblasts, was upregulated from day 3 post-BMP9-stimulation, with peak expression on day 5 (Fig. 1B). This pattern of expression of Runx2 negatively correlated with the early decrease in miR-17 expression but did not correlate with later miR-17 expression. Similar results were seen in the expression pattern of the late osteogenic marker osteocalcin (OCN). Taken together, these results suggest that miR-17 affects the BMP9-induced osteogenic differentiation that precedes the full commitment of MSCs to an osteogenic lineage.

### miR-17 overexpression inhibits BMP9-induced osteogenic differentiation in vivo and in vitro

We conducted overexpression experiments to determine the effect of individual members of the miR-17-92 cluster on BMP9-induced osteogenic differentiation of MSCs. We generated separate stable iMEF cell lines overexpressing miR-17 (miR-17 iMEFs), miR-18a (miR-18a iMEFs), miR-20a (miR-20a iMEFs) and miR-92a-1 (miR-92a-1 iMEFs), and a control cell line (pMPB iMEFs, hereinafter referred to as miR Control iMEFs) (Fig. 1C). Using qPCR analysis, we demonstrated that the PiggyBac system was able to effectively upregulate the expression of miR-17-92 cluster members (Fig. 1D).

**Fig. 2:**
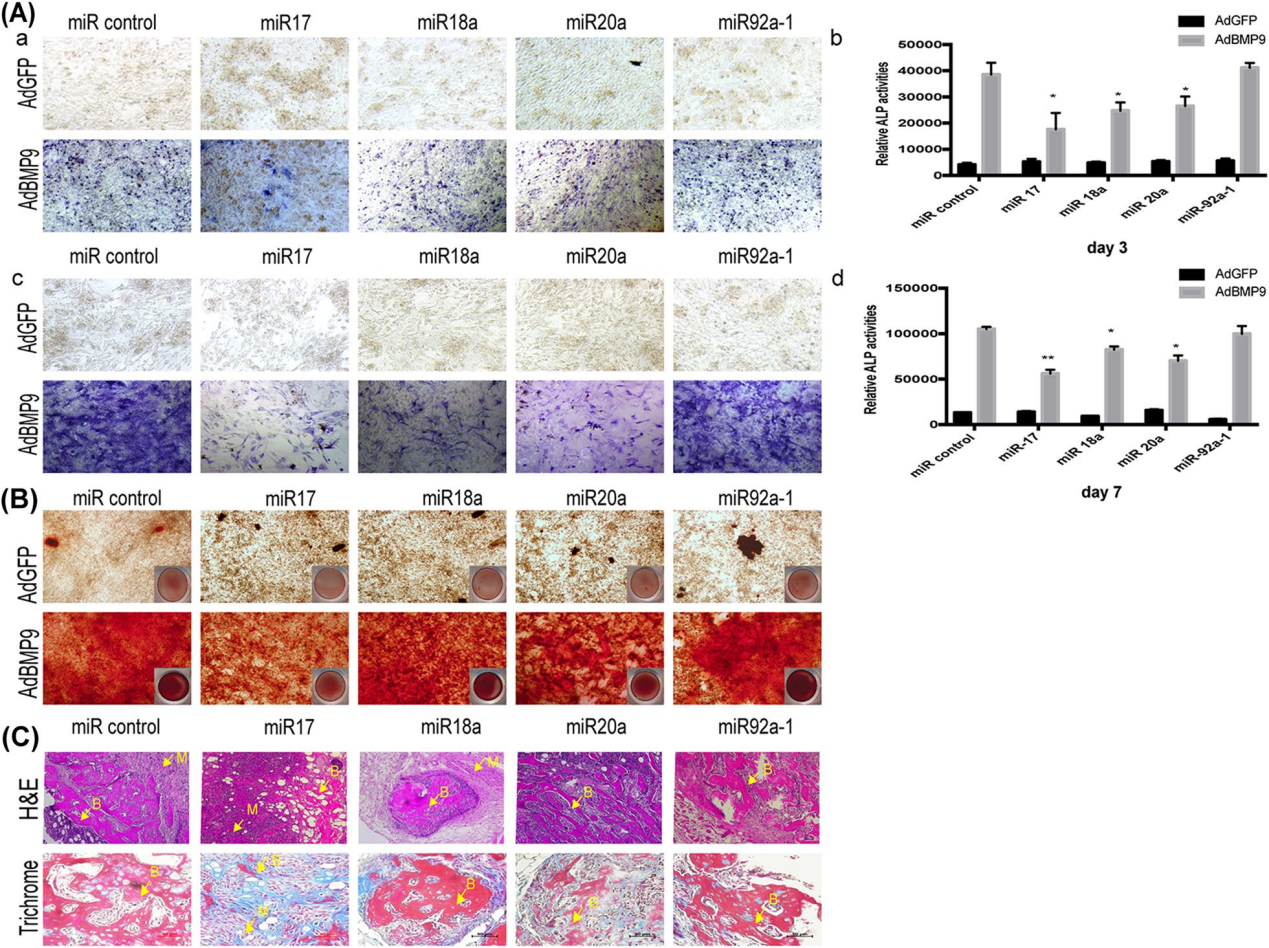
The osteogenic effect of miR-17-92 cluster on BMP9-induced osteogenesis in vitro and in vivo. **A** The individual member of miR-17-92 cluster in BMP9-induced ALP activity in MSCs. Sub-confluent miR-17, miR-18a, miR-20a and miR-92a-1 iMEFs were infected with AdBMP9 or AdGFP at day 3 (a, b) and day 7 (c, d). Histochemical staining (a, c) and quantitative bioluminescence assay (b, d). Each assay was done in triplicate. **p < 0.01. **B** The function of individual member of miR-17-92 cluster in BMP9-induced calcium deposit. The infected cells were fixed and subjected to Alizarin Red S staining at day 14. Microscopic images (10 ×) are shown. **C** H & E (a) and Masson’s Trichrome staining (b) of the bone masses retrieved at week 4. B, mature bone; M, undifferentiated MSCs

We then analyzed the effect of the upregulated expression of the miR-17-92 cluster members on BMP9-induced osteogenic differentiation. miR-17 iMEFs, miR-18a iMEFs, miR-20a iMEFs, miR-92a-1 iMEFs, and miR Control iMEFs were infected separately with either AdBMP9 or AdGFP. ALP activity, a marker of early osteogenesis, was then measured. We found that both qualitative and quantitative ALP activity was significantly decreased on day 3, and, to a lesser extent, on day 7 in the miR-17 iMEFs, miR-18a iMEFs, and miR-20a iMEFs (Fig. 2A). Furthermore, BMP9-induced matrix mineralization in the iMEFs was significantly decreased on day 14 in the miR-17 iMEFs, whereas no significant change was seen in either the miR-18a iMEFs, miR-20a iMEFs, or miR-92a-1 iMEFs (Fig. 2B). Thus, overexpression of miR-17 inhibits the entire period of BMP9-induced osteogenic differentiation, while overexpression of miR-18a and miR-20a only inhibits the early stage. Collectively, these results strongly suggest that miR-17 plays a pivotal role in the regulation of BMP9-induced osteogenic differentiation.

To further investigate the function of the miR-17-92 cluster members in the BMP9-induced osteogenic differentiation, in vivo assays were performed. Our results indicate that miR-17 dramatically inhibits this process. H&E staining of the retrieved masses further confirmed that overexpression of miR-17 significantly decreased BMP9-induced osteogenesis, evidenced by the diminished formation of trabecular bone and/or osteoid matrix, compared with the miR control iMEFs. Furthermore, trichrome staining demonstrated that the masses retrieved from the miR-17 iMEFs contained scant mineralized and mature osteoid matrix, compared with that of miR Control iMEFs (Fig. 2C). Altogether, both our in vivo and our in vitro results indicate that miR-17 overexpression inhibits BMP9-induced osteogenic differentiation, suggesting that maintaining an appropriate expression level of miR-17 may be critical for successful BMP9-induced osteogenic differentiation.

### A miR-17 sponge rescues the osteogenic impairment caused by miR-17 overexpression in vitro and in vivo

To further investigate whether endogenous miR-17 functions as an inhibitor of BMP9-induced osteogenic differentiation, we designed an antagomiR sequence against miR-17 (a miR-17 sponge). AntagomiRs act as molecular sponges and silence endogenous miRNAs.

**Fig. 3:**
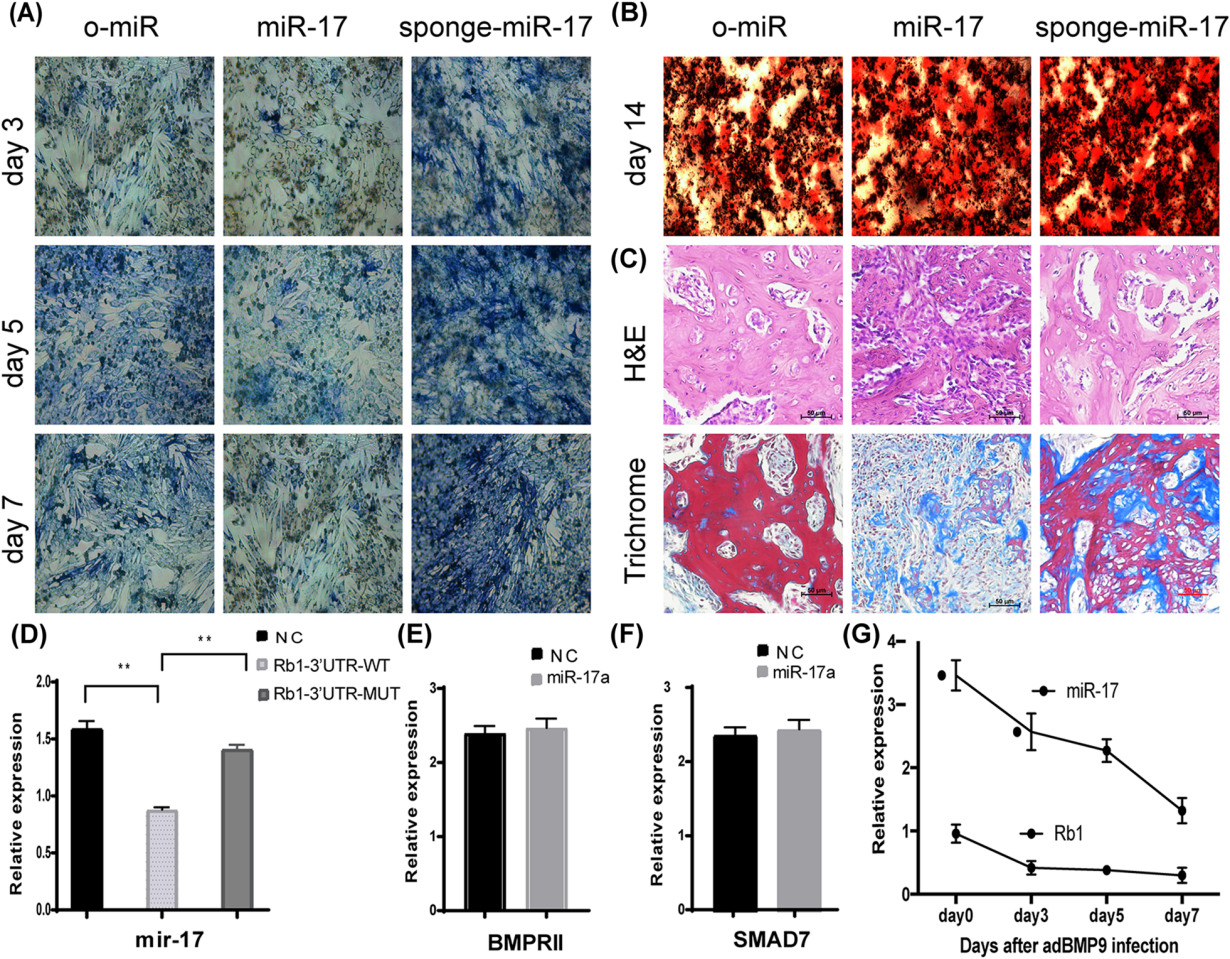
miR-17 inhibits BMP9-induced ectopic bone formation in vivoand in vitro*.* Sponge miR-17 rescued down-regulated ALP activity at day 3,5, and 7. (**A**) and calcium deposit at day 14 (**B**) in BMP9-induced miR-17 iMEFs. **C** H & E (a) and Masson’s Trichrome staining (b) of the bone masses retrieved at week 4. B, mature bone; M, undifferentiated MSCs. iMEF cells were co-transfected with the luciferase reporters carrying 3′UTR of wild-type or mutated *Rb1* (**D**), *BMPRII* (**E**) and *SMAD7* (**F**). **G** Expression levels of miR-17 and *RB1* in BMP9-induced osteogenic differentiation of MSCs

We found that overexpression of the miR-17 sponge in miR-17 iMEFs led to significant recovery of both qualitative and quantitative BMP9-induced ALP activity on days 3, 5, and 7 (Fig. 3A). Moreover, overexpression of the miR-17 sponge significantly increased BMP9-induced matrix mineralization on day 14 (Fig. 3B).

In vivo assays showed that overexpression of miR-17 sponge in miR-17 iMEFs dramatically increased BMP9-induced osteogenic differentiation. H&E and trichrome staining of the retrieved masses further confirmed this finding: overexpression of the miR-17 sponge led to increased formation of trabecular bone and/or osteoid matrix compared to miR-17 iMEFs without miR-17 sponge overexpression (Fig. 3C).

Overall, these results show that inhibition of endogenous miR-17 in vitro and in vivo using the miR-17 sponge significantly increases BMP9-induced osteogenic differentiation.

### miR-17 binds to the 3′-UTR of the RB1 gene to downregulate the expression of RB1

Potential miR-17 target genes were identified using a bioinformatics approach. TargetScan (http://genes.mit.edu/targetscan) software predicted three target genes for miR-17: *RB1*, *BMPR2*, and *SMAD7* (Additional file 1: Fig. S1). These genes all play important roles in osteogenesis. Using a luciferase reporter assay, we showed that miR-17 inhibited the luciferase activity of the 3′-UTR of *RB1*, but not the 3′-UTRs of mutated *RB1*, *BMPR2*, or *SMAD7* (Fig. 3D). These results suggest that *RB1* is a direct target of miR-17.

Because of the importance of *RB1* in osteoblastic differentiation [25], we studied the expression patterns of both miR-17 and *RB1* during BMP9-induced osteogenic differentiation of iMEFs. TqPCR was used to measure the expression of miR-17 and *RB1* at different timepoints during the osteogenic differentiation process. The expression pattern of *RB1* was negatively correlated with that of miR-17: *RB1* was highly expressed in cells with low miR-17 levels, whereas *RB1* expression was downregulated in cells with high miR-17 levels (Fig. 3E).

### *RB1* is essential for miR-17 effects on osteogenesis in vitro and in vivo

**Fig. 4:**
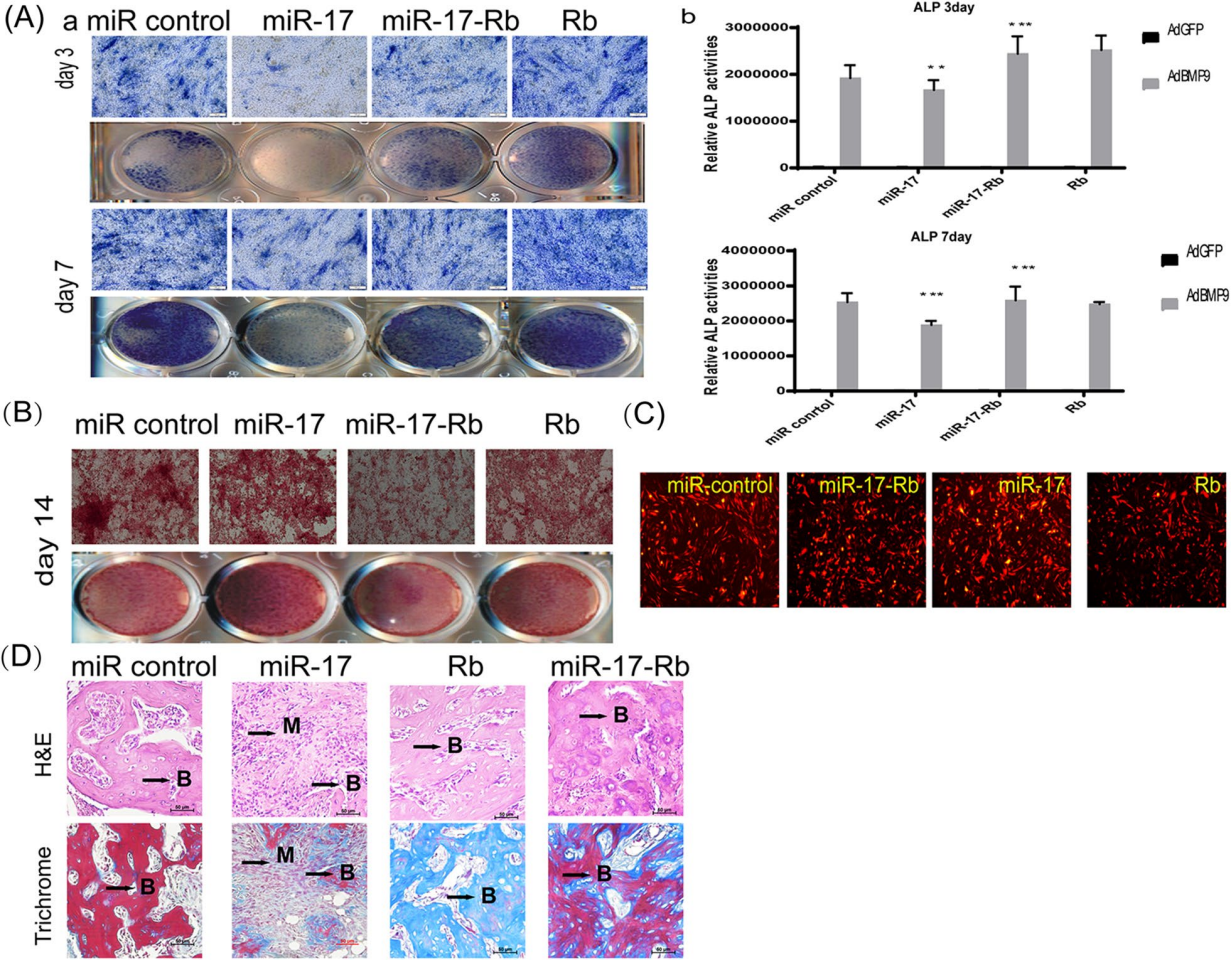
RB1 is essential for miR-17 effect on osteogenesis in vivo and in vitro. **A** RB1 rescued BMP9-induced ALP activity in miR-17 iMEFs. Qualitative histochemical staining (a) and Quantitative bioluminescence assay (b). **p < 0.01. **B** RB1 rescued BMP9-induced calcium deposit in miR-17 iMEFs. **C** Adenovirus adBMP9 overexpression in stable cell lines. **D** H & E (a) and Masson’s Trichrome staining (b) of the bone masses retrieved at week 4. Representative images are shown. B, mature bone; M, undifferentiated MSCs

We performed both in vitro and in vivo experiments to further investigate whether miR-17 negatively regulated BMP9-induced osteogenesis through the *RB1* gene. Using the PiggyBac system, we overexpressed *RB1* in iMEFs (to create iMEF-Rb1 cells) and miR-17 iMEFs (to create miR-17 iMEFs-Rb1 cells) and investigated the BMP9-induced osteogenic differentiation of MSCs. We found that the BMP9-induced ALP activity was significantly inhibited on days 3(Fig. 4A a,b) and, to a lesser extent, on day 7 (Fig. 4c,d). We also found that the BMP9-induced matrix mineralization in the iMEFs was significantly inhibited by *RB1* overexpression on day 14 (Fig. 4B).

For the in vivo stem cell implantation studies, we used the miR control iMEF, iMEF-Rb1, miR-17 iMEFs, and miR-17 iMEFs-Rb1 stable cell lines; the *RB1* gene expression of these cell lines is shown in Fig. 4C. The cell lines were transduced with AdBMP9 or AdRFP, then subcutaneously injected into the flanks of athymic nude mice. We found that, after 4 weeks, bony masses could be retrieved from the injection sites of the AdBMP9 treatment groups, while no masses were retrieved from the injection sites of the AdGFP-treated miR control groups. H&E staining of the retrieved masses confirmed that expression of either *RB1* alone or *RB1* combined with the overexpression of miR-17 significantly increased BMP9-induced bone formation, as evidenced by an increased formation of trabecular bones and/or osteoid matrix, compared with that of the miR-17 iMEFs group. Trichrome staining further demonstrated that the masses retrieved from both the iMEF-Rb1 and miR-17 iMEFs-Rb1 groups exhibited increased mineralized and mature osteoid matrix, compared with that of the miR-17 iMEFs group (Fig. 4D). Collectively, these in vivo results suggest that exogenous expression of *RB1* may rescue the diminished terminal osteogenic differentiation caused by the overexpression of miR-17 in MSCs.

Taken together, these in vitro and in vivo results strongly suggest that miR-17 modulates the BMP9-induced osteogenesis of MSCs, and that this modulation can be reversed by its target gene *RB1*.

## Discussion

In this study, we provide both in vitro and in vivo evidence that miR-17 inhibits osteogenic differentiation in iMEFs, our MSC lines. We also investigated three other members of the miR17-92 cluster (miR-18a, miR-20a, and miR-92a-1) and found that they did not play a significant role in BMP9-induced osteogenesis, although the miR17-92 cluster spans only 800 bp, and the members share similar seed regions. This phenomenon of members of a miRNA cluster exhibiting unique effects may be due to spatiotemporal expression heterogeneity [28].

Bone remodeling, the process of destruction and synthesis that gives bone its mature structure, is an essential and necessary process for bone regeneration [29]. Imbalances in bone remodeling have been considered etiological factors in several skeletal diseases, most notably osteoporosis [30]. Because of their ability to differentiate into multiple lineages, MSCs have been extensively studied as candidates for tissue engineering and regenerative medicine [31]. There is also growing interest in the role of miRNAs in MSC differentiation. In this study, we found that overexpression of miR-17 inhibits BMP9-induced osteogenesis and downregulates osteogenesis-related genes. Moreover, we found that a miR-17 sponge, which silenced the endogenous miR-17, had positive effects on the osteogenic process, as it increased the levels of osteogenesis-related genes. In vivo experiments also showed that miR-17 inhibited BMP9-induced osteogenesis. Collectively, our findings suggest that miR-17 is a negative regulator of osteogenesis. A single miRNA usually targets multiple genes. Consequently, the expression patterns of the target genes contribute to the cell- or tissue-specific functions of that particular miRNA. Several genes in different tissues have been proposed as potential target genes for miR-17. For example, miR-17 has been shown to target genes involved in normal biological processes (such as neurogenesis) as well as genes implicated in diseases (e.g., cancer and diabetes mellitus) [32–36]. Our study results demonstrate that miR-17 inhibits the expression of the *RB1* gene by binding to partially complementary sequences in the 3′-UTR. Interestingly, *RB1* expression was negatively correlated with miR-17 expression during osteogenesis, suggesting that *RB1* is indeed a downstream target gene of miR-17. The *RB1* gene is located at the q14 region of human chromosome 13 (13q14.2). *RB1* produces a 4.8-nt mRNA, within which a 2.7-nt region encodes the retinoblastoma tumor suppressor protein (pRb) [37]. Recent studies have shown that pRb contributes to osteoblast differentiation and bone development [38]. Moreover, the incidence of osteosarcoma is increased 500-fold in patients with a pRb deficiency caused by mutation in the *RB1* gene. pRb physically interacts with core-binding factor subunit alpha-1 (CBFA1), an osteoblast transcription factor, and functions as a direct transcriptional coactivator that promotes osteoblast differentiation [39]. This may be the reason why pRb knockout mice generally develop bone abnormalities: without pRb, osteoblasts fail to assemble cell-to-cell adheres junctions [40]. Berman et al. suggested that pRb plays a key role in regulating osteoblast differentiation by inhibiting E2F1, a member of the E2F family of transcription factors [41]. Consistent with these results, our study shows that RB1 accelerates osteoblast differentiation both in vitro and in vivo.

## Conclusion

Our study shows that miR-17, a member of the miR17-92 cluster, inhibits both in vitro and in vivo BMP9-induced osteogenic differentiation. We also demonstrate that *RB1* is a direct target gene of miR-17 and that overexpression of *RB1* attenuates the inhibitory effects of miR-17 on the osteogenic process. These results are clinically relevant, as anti-miRNA therapies directed to inhibit miR-17 could be used to increase the allocation of MSCs undergoing osteogenic differentiation, ultimately increasing the bone regenerative capacity.

## Supplementary Information

**Additional file 1: Fig. S1.** Schematic of miR-17 putative target site in 3′UTR of mouse *Rb1* (A), *BMPRII* (B) and *SMAD7* (C). Alignment of miR-17 with wild-type (WT) and mutant (MUT) 3′UTR region of *Rb1*, *BMPRII* and *SMAD7* showing complementary pairing. The 3′ mutated nucleotides are underlined.

## Data Availability

We state that the data will not be shared since all the raw data are present in the figures included in the article.

## Abbreviation

BMP9: Bone morphogenetic protein 9
MSCs: Mesenchymal stem cells
miRNAs: MicroRNAs
UTR: Untranslated region
RB1: Retinoblastoma gene
MSCs: Mesenchymal stem cells
ncRNAs: Non-coding RNAs
BMPs: Bone morphogenetic proteins
iMEFs: Immortalized mouse embryonic fibroblasts
DMEM: Dulbecco's Modified Eagle’s Medium
FBS: Fetal bovine serum
TqPCR: Quantitative real-time PCR
ALP: Alkaline phosphatase
Runx2: Runt-related transcription factor 2
OCN: Osteocalcin
