# Supplementary figures and images for: Investigation of the role of the miR17-92 cluster in BMP9-induced osteoblast lineage commitment
Source: J Orthop Surg Res. 2021 Oct 30;16:652. doi: 10.1186/s13018-021-02804-9 (PMC8557618; doi:10.1186/s13018-021-02804-9)

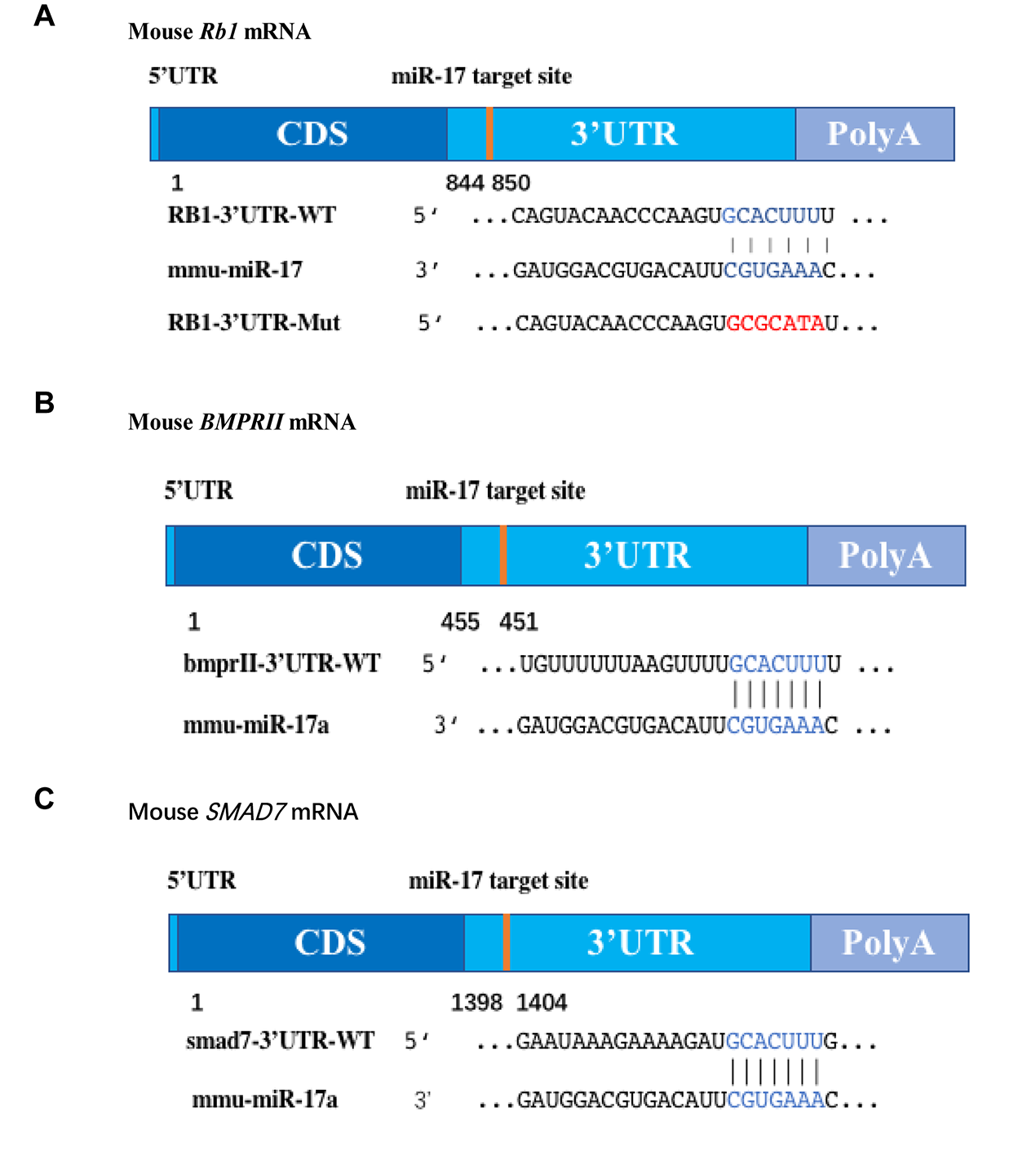

Supplement: Supplementary file 1 — Additional file 1: Fig. S1. Schematic of miR-17 putative target site in 3′UTR of mouse Rb1 (A), BMPRII (B) and SMAD7 (C). Alignment of miR-17 with wild-type (WT) and mutant (MUT) 3′UTR region of Rb1, BMPRII and SMAD7 showing complementary pairing. The 3′ mutated nucleotides are underlined. [file 13018_2021_2804_MOESM1_ESM.tif]
